# Reestablishment of the Smile after Hypoglossal–Facial Nerve Transfer: What Can We Learn?

**DOI:** 10.1055/a-2128-5191

**Published:** 2023-08-11

**Authors:** M.C. Kleijwegt, C. Wever, E.F. Hensen, J.C. Jansen, R.W. Koot, M.J.A. Malessy

**Affiliations:** 1 ENT Department, Leiden University Medical Center, Leiden, The Netherlands; 2 Neurosurgery Department, Leiden University Medical Center, Leiden, The Netherlands

**Keywords:** vestibular schwannoma, facial reconstruction, hypoglossal, nerve transfers, facial schwannoma

## Abstract

**Objective** The aim of this study was to assess the ability to smile following a hypoglossal–facial nerve transfer (N12–N7).

**Design** This is a retrospective chart review.

**Setting** National tertiary referral center for skull base pathology.

**Participants** Seventeen patients.

**Main Outcome Measures** The ability to smile following an N12–N7 transfer was assessed by five medical doctors on photographs of the whole face and frontal, orbital, and oral segments. The (segmented) photographs were scored for the symmetry, asymmetry, and correct or incorrect assessment of the affected side.

**Results** Seventeen patients were analyzed by 5 assessors providing 85 assessments. The whole face at rest was judged symmetrical in 26% of the cases and mildly asymmetrical in 56%. Frontal, orbital, and oral segments were symmetrical in 63, 20, and 35%, respectively. The affected side was correctly identified in 76%. When smiling, the whole face was symmetrical in 6% and mildly asymmetric in 59%. The affected side was correctly identified in 94%. The frontal, orbital, and oral segments during smiling were symmetrical in 67, 15, and 6%, respectively. The affected side of the frontal, orbital, and buccal facial segments during smiling was correctly identified in 89, 89, and 96%, respectively. Interobserver variability with Fleiss' kappa analysis showed that the strength of the agreement during smile of the total face was good (0.771)

**Conclusion** Following an N12–N7 transfer, a good facial symmetry at rest can be achieved. During smiling, almost all patients showed asymmetry of the face, which was predominantly determined by the orbital and oral segments. To improve the ability to smile after an N12–N7 transfer, additional procedures are needed.

## Introduction

Facial paralysis caused by facial nerve (N7) lesion due to trauma or surgery is a devastating condition that may result in lifelong loss of function of the muscles in the frontal, orbital, and oral segments, affecting the ability to frown and close the eye and mouth. In addition, the quality of life is diminished by the loss of the ability to express emotion through smiling. 1 2 3 4

In the past decades, different techniques for facial nerve reconstruction have been proposed. 3 5 6 7 8 9 10 One of these is the hypoglossal–facial nerve (N7–N12) transfer, of which numerous technical modifications have been described. 10 One of them is the partial (hemi) use of the hypoglossal nerve with direct end to side coaptation to reduce hemiatrophy of the tongue and diminish recovery time. 2 9 11 12 13

Over the years, many systems to grade the facial nerve function have been developed. 14 15 Historically, the House–Brackmann (H-B) score is the most well-known and widely used grading system to score facial nerve function, using both characteristics at rest and in motion. 16 Although originally not developed to score the facial function after reconstruction and despite its shortcomings, the H-B grading system is also frequently used in studies reporting on the outcome of the N7–N12 transfer, namely, in around 70%. 17 The H-B grading does not clearly differentiate between the function of different segments of the face at rest and in an active phase. Therefore, detailed information about the potential differences between the function in a static or dynamic phase, for instance smiling, is limited. 14 We know that a good smile suggests increased intelligence, happiness, and social status. Therefore, smiling is fundamental in facial reanimation. 4

In this study, we evaluate our results of facial reconstruction using the N7–N12 transfer and specifically focus on the ability to smile. Five medical doctors blinded for the side of the N7–N12 transfer independently assessed photographs of the whole face at rest and while smiling. Additionally, the photographs were divided in three segments (frontal, orbital, and oral) to determine to what extent it is possible to generate a smile following an N7–N12 transfer.

## Material and Methods

### Patients

In this retrospective cohort study, patients who underwent an N7–N12 nerve transfer between 2001 and 2019 were included. Sixteen patients had an N7 lesion following skull base surgery and one patient after cholesteatoma surgery (mastoidectomy). We consider the N7–N12 nerve transfer as the best first step in facial reanimation in this situation as it potentially reinnervates all muscles of the face. Clinical data were collected from medical records, including the cause of the loss of facial nerve function, interval between facial paralysis and surgical reconstruction, outcome of H-B grading, and complications during reconstruction. 18

Patients were excluded when (1) the follow-up was less than 1 year; (2) the facial nerve deficit occurred following resections of malignant tumors; (3) postoperative photographs were unavailable; and (4) major static procedures were additionally performed (e.g., forehead lift).

**Fig. 1:**
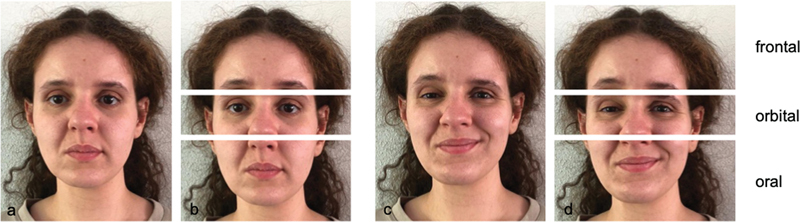
of photographs and segments ( **a,b** ) at rest and ( **c,d** ) in the active phase.

Digital photographs of the entire face were made by a clinical photographer, and if not present, they were provided by the patients following instructions. The patients were asked to keep the face at rest and to smile to the best of their ability as they would normally do. The photographs of the entire face were digitally divided into three segments: frontal, orbital, and oral ( Fig. 1 ). The boundaries of the orbital segment were just cranial and caudal to the supra- and infraorbital margins covering the area of the orbicularis oculi muscle. The frontal segment was the part cranial to the orbital segment, and the oral segment was the part caudal to the orbital segment.

All the segmented photographs at rest and while smiling were mixed at random and separated from those of the entire face.

The photographs were assessed by five medical doctors individually (2 neurosurgeons, 2 ear, nose, and throat [ENT] surgeons, and 1 ENT resident) who were blinded to the side of the N12–N7 transfer. First, the segmented photographs were assessed, and 1 week later those of the entire face. The assessors were asked to indicate whether the face was symmetrical or asymmetrical and if asymmetrical to identify the affected side. If the face was asymmetrical, they had to score whether it was mildly or severely disfiguring. The identification of the affected side was compared with the clinical data, and defined as correct or incorrect.

This study was evaluated by the medical ethics committee of the Leiden University Medical Center (LUMC). The committee judged that medical ethical review was not required because of the retrospective nature, and patients were not subjected to any procedures and/or behavioral restriction.

### Surgical Technique of N12–N7 Transfer

The surgical technique applied was extensively described previously. 2 In short, the extratemporal portion of the facial nerve was identified via a parotid incision, using the posterior belly of the digastric muscle and tragal pointer. The vertical part of the facial canal in the mastoid bone was unroofed. The intratemporal part of the facial nerve was mobilized and transected at the external facial nerve (second) genu. The hypoglossal nerve was identified at the level of the carotid bifurcation and neurolyzed as proximally as possible. The hypoglossal nerve was partially cut such that the exposed area corresponded to the cross-sectional area of the facial nerve. A tensionless end-to-side coaptation between the two nerves was made using 10–0 sutures and glue.

### Statistical Analysis

Statistical analysis was performed using GraphPad Prism (version 9). The assessments in which the affected side was correctly identified were tested using Fisher's exact test for a comparison between the assessments of the total face at rest and in the active phase (smiling). For the segmented assessments, this was done with the chi-squared test. The hypothesis was that the observer can identify the affected side more accurately in a smiling patient. The interobserver variability was scored using the Fleiss kappa analysis in IBM SPSS Statistics version 28.0.1.0. The following strength of agreement was used: less than 0.20 poor, 0.21 to 0.40 fair, 0.41 to 0.60 moderate, 0.61 to 0.80 good, and 0.81 to 1.00 very good. 19 Pre- and postoperative H-B grading was tested for significance. A *p* -value of less than 0.05 was considered significant.

## Results

**Fig. 2:**
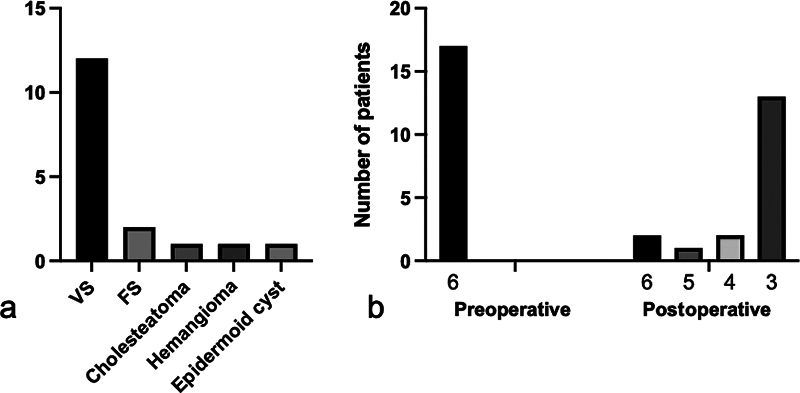
( **a** ) Overview of different pathologies per number of patients resulting in facial nerve deficit. VS, vestibular schwannoma; FS, facial schwannoma. ( **b** ) Pre- and postoperative House–Brackmann classification.

The study comprised 11 female and 6 male patients with a mean age of 43.5 years at the time of facial paralysis (SD ± 17.7; range: 8–68; median: 44; Table 1 ). All the patients had a facial paralysis following surgery for vestibular schwannoma (VS; *n* = 12), facial schwannoma ( *n* = 2), hemangioma ( *n* = 1), epidermoid cyst ( *n* = 1), and cholesteatoma ( *n* = 1; Fig. 2a ). The average interval between facial paralysis and reconstructive surgery was 5.2 months (SD ± 4.6; range: 0–15; median: 4). Four patients with preserved facial nerve continuity during VS resection had a longer interval (average: 10 months) as compared with the overall average, reflecting the time that passed to assess whether potential spontaneous recovery would occur. Two patients had surgery and/or facial nerve reconstructions elsewhere (patient nos. 3 and 5) and were reconstructed late (>12 months). In one of these patients (no. 5) initially an end-to-end coaptation of the facial nerve was performed and in the second instance the N7–N12 transfer was performed. All patients scored H-B VI prior to the N12–N7 transfer. No complications occurred following the N12–N7 surgery. The H-B grading was performed during outpatient visits with a mean postnerve transfer interval of 62.5 months (SD ± 49.8; range: 17–172; median: 40). Postoperatively, 13 patients improved to H-B grade III (76%), 1 patient to grade IV (6%), 1 to grade V (6%), and in 2 patients facial function did not recover, with a persisting H-B grade VI (12%; Table 1 , Fig. 2b ). Patients 3 and 5, who underwent a reconstruction late had postoperative H-B grade VI (no. 3) and IV (no. 5). Of the two facial schwannoma patients, one had postoperative H-B grade V and one had a postoperative H-B grade VI. A gold weight was inserted in the upper eyelid of the affected side to improve closure in three patients. Tarsorrhaphies were performed in three patients. Four patients had synkinesis, which was treated with botulinum toxin. None of the patients perceived loss of function of the tongue.

**Table 1:** Results and patient characteristics of the patients who underwent a hypoglossal to facial nerve transfer

Patient no.	Age (y)	Gender	Pathology	Preoperative HB	Postoperative HB	Interval lesion-surgery (mo)	FU (mo)
1	43	F	VS a	6	3	4	38
2	63	F	VS	6	3 b	1	117
3	18	F	VS a	6	6 c d	15	172
4	37	F	VS	6	3 b c	4	128
5	8	M	Cholesteatoma	6	4 c	13	93
6	21	F	VS	5	3	2	20
7	51	F	VS	6	3	2	21
8	68	M	VS	6	3 d	1	59
9	37	M	FS	6	5 d	5	22
10	57	F	VS	6	3	11	26
11	44	M	VS a	6	3	7	80
12	55	M	Hemangioma	6	3 b	2	40
13	43	F	VS	6	3	2	117
14	63	M	FS	6	6	4	18
15	20	F	Epidermoid cyst	6	3	7	17
16	55	F	VS a	6	3	9	19
17	55	F	VS	6	3 b	2	94

Abbreviations: F, female; FS, facial schwannoma; FU, routine follow-up in months; HB, House–Brackmann; M, male; VS, vestibular schwannoma.

a Neuropraxia.

b Botulinum toxin injections.

c Gold weight eyelid.

d Tarsorrhaphy.

Postoperative photographs were made with a mean interval of 93 months after the N12–N7 reconstruction (SD ± 67.3; range: 12–212; median: 108). In total, 85 assessments of photographs at rest and during smile were performed. For the frontal, orbital, and oral segments, 255 assessments (17 patients, 3 segments, and 5 accessors) both at rest and during smile were performed.

**Fig. 3:**
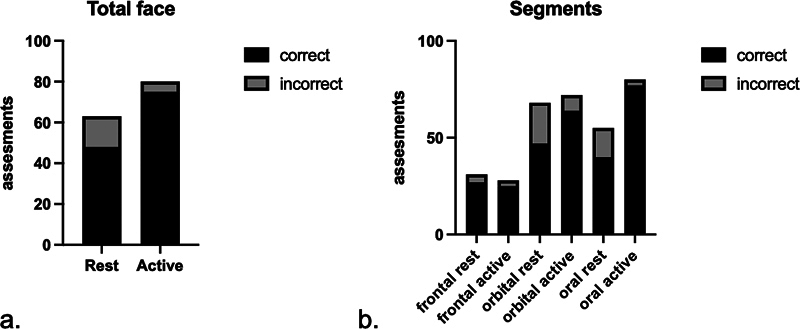
( **a** ) Results of photographical analysis of the total face, showing the proportion of correct and incorrect scores by the five medical observers. A total of 17 patients were scored by five observers. Fisher's exact test shows a significant difference between the two phases. ( **b** ) Results of the photographical analysis of three segments, showing the proportional correct versus incorrect per segment at rest versus the active phase. The chi-squared test shows a significant difference in all three segments.

The results of the photographic analyses are shown in Fig. 3 and Tables 2 , 3 , 4 . The total face at rest was symmetrical in 22 of 85 (26 %) patients. The affected side was significantly less well identified at rest as compared with during smiling (48/63 vs. 75/80; *p* = 0.003; Fig. 3a ). Asymmetry ( *n* = 63) was judged as mildly disfiguring in 48/63 (76%) of the patients. When smiling, asymmetry was scored as severely disfiguring in 30/80 (38%) patients, which was 15/63 (24%) patients at rest.

**Table 2:** Symmetry assessment of the entire face at rest and during smile (active) following hypoglossal to facial nerve transfer ( *n* = 85). Correct identification of the affected side was only scored with asymmetry

	Symmetry	Asymmetry	Correct identification
Rest	22 (26%)	63 (74%)	48 (76%)
Active	5 (6%)	80 (94%)	75 (94%)

**Table 3:** Symmetry and level of disfigurement in photographical analysis of the entire face following a hypoglossal to facial nerve transfer, at rest and during smile (active), *n* = 85

	Symmetry	Disfigurement	
		Mild	Severe
Rest	22 (26%)	48 (56%)	15 (18%)
Active	5 (6%)	50 (59%)	30 (35%)

**Table 4:** Assessment of the frontal, orbital, and oral segment photographs

		Symmetry, *n* (%)	Asymmetry, *n* (%)	Correct, *n* (%)
Frontal	Rest	54 (64)	31 (36)	27 (87)
	Active	57 (67)	28(33)	25 (89)
Orbital a	Rest	17 (20) a	68 (80)	47 (69)
	Active	13 (15) a	72 (85)	64 (89)
Oral	Rest	30 (35) a	55 (65)	40 (73)
	active	5 (6) a	80(94)	77 (96)

a Significant difference between the orbital and oral symmetrical segments ( *p* = 0,012) using Fisher's exact test. Correct identification of the affected segment was only scored in asymmetric faces.

In the analysis of the segments, the oral segment during smiling was asymmetrical in 80 of 85 (94%) patients. At rest, the oral segment was symmetrical in 30 of 85 (35%) patients. The affected side in the oral segment during smiling was correctly identified in 77 (96%) patients. The frontal segment was symmetrical at rest in 54 (64%) patients and during smiling in 57 (67%) patients. The orbital segment during smiling was asymmetrical in 72/85 (85%) patients. In the rest phase, the orbital segment was symmetrical in 17/85 (20%) patients. There was a significant difference ( *p* = 0.012) between the orbital and oral segments regarding symmetry at rest and during smiling ( Table 4 ). The identification of the affected side in the active phase in all three segments differed significantly from the rest phase ( *p* < 0.0001; Fig. 3b ).

The results of the interobserver variability using the Fleiss kappa analysis showed that the level of agreement during smile was good in the total face (0.771) and in the oral segment (0.641) and moderate in the orbital segment (0.420; Table 5 ).

**Table 5:** Interobserver variability scores using Fleiss' kappa analysis

Assessment	Intraclass correlation (kappa)	95% confidence interval	
		Lower bound	Upper bound
Rest total face	0.167	0.057	0.277
Active total face	0.771	0.636	0.906
Score of total face, rest	0.215	0.105	0.326
Score of total face, active	0.363	0.234	0.491
Rest frontal segment	0.313	0.219	0.406
Rest orbital segment	0.134	0.031	0.238
Rest oral segment	0.326	0.217	0.436
Active frontal segment	0.206	0.110	0.302
Active orbital segment	0.420	0.307	0.533
Active oral segment	0.641	0.513	0.769

## Discussion

The N12–N7 transfer is widely used to treat the sequelae of a facial nerve lesion. However, not much is known about the functional recovery of facial muscles. This is due to the fact that the grading systems that have been applied in the reports on outcome of the N12–N7 transfer do not clearly differentiate between the face at rest and during an active phase, such as smiling. In this study, we found that during smiling, professionals could correctly assess the affected side in more than 90% of cases. There was a 20% increase of asymmetry between rest and smile which was mainly caused by the oral segment of the face and to a lesser extent by the orbital region. These findings suggest that application of additional dynamic procedures to improve the oral segment may be a logical first step to improve smiling after the N12–N7 transfer.

The affected side with the face at rest was correctly identified in only 76%. Apparently, it was not evident to distinguish which side of the face was normal and which was reinnervated by the N12–N7 transfer. This might indicate that the appearance of what was mistakenly perceived as the unaffected side, but actually was the N12–N7 reinnervated side, cannot be grossly abnormal. However, the N12–N7 transfer results in a combination of flaccid paralysis components mixed with synkinetic activity, superimposed on faces that may also demonstrate normal aging phenomena.

In this study, we asked the patients to smile as they would normally do to the best of their ability. Providing these instructions did not lead to an active smile. It is worth noting that patients can generate a smile following an N12–N7 transfer. To do so, they have to consciously and forcefully push the tongue against the hard palate. Thereby, the original motor program of the tongue is used to activate the facial muscles. Apparently, the central program to activate a spontaneous smile does not activate hypoglossal motoneurons, which would require central plastic changes to occur. 20

The insufficient activity of the oral segment after the N12–N7 transfer may be improved by additional static or dynamic techniques. 4 7 8 10 One option that we currently use is to combine the N12–N7 transfer with a transfer of the masseteric nerve branch (N5, trigeminal nerve) to the oral branch of the facial nerve. An N5–N7 transfer alone does not provide symmetry at rest as good as the N12–N7 transfer. Therefore, a combination might prove optimal. 17 To create a smile after an N5–N7 transfer, however, one has to close the jaw. Although this is also different from spontaneous smiling, this action comes closer to a natural smile. Additionally, clenching the teeth to smile is easier to perform than pushing the tongue against the palate.

Cross-facial nerve grafting is another option to reanimate the facial musculature. If the facial musculature is withered, it is one of the very few options, but should be accompanied with a gracilis muscle transfer to regain dynamic function. This technique provides a positive trend in disease-specific quality of life. 21 However, the cross-facial technique is complex and requires multiple surgeries, each of which has failure rates. These factors have to be taken into consideration when choosing the best type of treatment, and these should be discussed with the patients to achieve optimal shared decision-making.

In a previous study of our group, we reported the outcome of the N7–12 transfer procedures using the H-B grading system. In that study, 86% of the patients had a H-B grade III in contrast to 76% in the present study. 2 The difference can be explained by the fact that in our earlier report, patients with facial schwannomas were excluded. Facial schwannoma causes a slowly progressing paralysis, which usually takes years to develop. Irreversible atrophy of a part of the facial musculature occurs over time, excluding muscle fibers for reinnervation by a nerve transfer that causes a negative impact on the outcome. 22 23 24 If we exclude the patients with a facial schwannoma, the overall H-B grade III score in the remaining series increases to 87%, which is comparable with earlier reports. 6 11 Optimally, facial reinnervation following a complete injury right from the start is performed within 6 months after onset. 5 8 Since the process of facial nerve function deterioration is a gradual process in case of a facial nerve schwannoma, patient counseling with regard to the timing of nerve transfer is key for good outcome.

The weaknesses of this study are the relatively small number of patients, the fact that intraobserver variability was not assessed, and the retrospective nature of the study. The strength is that the outcome was independently performed by five assessors. In addition, the method of segmental analysis of the reconstructed (N12–N7) face provides deeper insight into the contribution of the frontal, orbital, and oral parts of the face to obtain symmetry and the generation of a smile. In our opinion, this study, using (segmented) photographs and observer assessments, is unique. Other studies concerning facial reanimation and postoperative results use different scoring systems, which are categorized in observational, mathematical, and computer-graphical measurements. 14 Nevertheless, this study addresses the question of whether an N7–N12 transfer generates a good smile, which was observed by five medical assessors.

## Conclusion

Following an N7–N12 transfer, the majority of patients achieve a good symmetry of the face at rest, but they are unable to generate a natural smile. Static and dynamic analysis of the facial nerve–innervated muscle function not only is essential to adequately evaluate the outcome of facial nerve reconstructions but also provides clues that additional dynamic procedures may be required to improve the overall outcome.
